# Molecular Evolution of the Primate Antiviral Restriction Factor Tetherin

**DOI:** 10.1371/journal.pone.0011904

**Published:** 2010-07-30

**Authors:** Jun Liu, Keping Chen, Jian-Hua Wang, Chiyu Zhang

**Affiliations:** 1 Institute of Life Sciences, Jiangsu University, Zhenjiang, Jiangsu, China; 2 Institut Pasteur of Shanghai, Chinese Academy of Sciences, Shanghai, China; University of Pittsburgh, United States of America

## Abstract

**Background:**

Tetherin is a recently identified antiviral restriction factor that restricts HIV-1 particle release in the absence of the HIV-1 viral protein U (Vpu). It is reminiscent of APOBEC3G and TRIM5a that also antagonize HIV. APOBEC3G and TRIM5a have been demonstrated to evolve under pervasive positive selection throughout primate evolution, supporting the red-queen hypothesis. Therefore, one naturally presumes that Tetherin also evolves under pervasive positive selection throughout primate evolution and supports the red-queen hypothesis. Here, we performed a detailed evolutionary analysis to address this presumption.

**Methodology/Principal Findings:**

Results of non-synonymous and synonymous substitution rates reveal that Tetherin as a whole experiences neutral evolution rather than pervasive positive selection throughout primate evolution, as well as in non-primate mammal evolution. Sliding-window analyses show that the regions of the primate Tetherin that interact with viral proteins are under positive selection or relaxed purifying selection. In particular, the sites identified under positive selection generally focus on these regions, indicating that the main selective pressure acting on the primate Tetherin comes from virus infection. The branch-site model detected positive selection acting on the ancestral branch of the New World Monkey lineage, suggesting an episodic adaptive evolution. The positive selection was also found in duplicated Tetherins in ruminants. Moreover, there is no bias in the alterations of amino acids in the evolution of the primate Tetherin, implying that the primate Tetherin may retain broad spectrum of antiviral activity by maintaining structure stability.

**Conclusions/Significance:**

These results conclude that the molecular evolution of Tetherin may be attributed to the host–virus arms race, supporting the Red Queen hypothesis, and Tetherin may be in an intermediate stage in transition from neutral to pervasive adaptive evolution.

## Introduction

To mitigate the susceptibility to various viruses (e.g. human immunodeficiency virus, HIV), primates have evolved innate cellular defense systems to inhibit virus replication in cells or virus release from cells. Up to now, three kinds of innate cellular defense systems have been identified [1], [2], [3], [4], [5]. Two of them are the antiviral restriction factors from APOBEC3 and TRIM families, and their antiviral mechanisms have been widely demonstrated during the past few years [6], [7]. However, Tetherin (also known as BST2, CD317 or HM1.24) that was recently identified as a novel antiviral restriction factor, remains some mysteries to be solved [8].

Tetherin is an interferon-inducible transmembrane protein. It was found because it can restrict the release of fully formed virus particles from infected cells in the absence of the HIV-1 viral protein U (Vpu) [4], [5]. When infecting certain human cell lines that contain Tetherin gene, such as HeLa, Vpu-deleted HIV-1 particles accumulate in endosomal vesicles and/or remain attached to the cell surface, leading to a failure in virus release from cell surface [9]. Although Tetherin-mediated virus retention may be independent of any viral protein target, it is antagonized by HIV-1 Vpu protein [10], [11]. Tetherin exhibits a wide spectrum of antiviral activity, including at least four virus families: retroviruses, filoviruses, arenaviruses, and herpesviruses. Accordingly, viral antagonists of Tetherin include HIV-1 Vpu, SIV Nef, HIV-2, SIV and Ebola envelope glycoproteins, and KSHV (Kaposi's sarcoma-associated herpesvirus) K5 protein [8], [11], [12]. Their antagonist mechanisms are involved in cellular endosomal trafficking pathway and ubiquitination-mediated protein degradation pathway [8].

Tetherin belongs to type II integral membrane proteins with an unusual topology. It contains a transmembrane anchor near its N-terminus that is located in the cytoplasm, followed by an extracellular coiled-coil domain and a putative glycophosphatidyl-inositol (GPI) anchor at its C-terminus [13]. In addition, it contains three conserved cysteines that are located in its extracellular region and are responsible for the formation of three conserved intermolecular disulfide bonds between Tetherin molecules. Therefore, Tetherin exists as a disulfide-bonded homodimer on cell surface [14]. The cysteine-mediated dimerization is very important in the restriction of HIV-1, but less important in the restriction of Lassa or Marburg virus [14], [15].

According to the red-queen hypothesis, host antiviral restriction factors (e.g. APOBEC3G and Tetherin) and viral countermeasures (e.g. Vif and Vpu) should engage in antagonistic coevolutionary arms races, which will result in rapid amino acid substitutions in both the proteins [16]. This hypothesis has been well demonstrated in the antagonism between human APOBEC3G and HIV-1 Vif, both which evolve rapidly by positive selection [17], [18], [19]. Similar to the interaction between APOBEC3G and Vif, the interaction between Tetherin and HIV Vpu is partially species-specific. For example, HIV Vpu obviously counteracts the Tetherins of human and chimpanzee, but not that from the mouse or African green monkey [20]. It implies that like APOBEC3G, primate Tetherin gene should also evolve under pervasive positive selection. Two recent studies showed that Tetherin has been under positive Darwinian selection and demonstrated that some positively selected sites influence Tetherin's sensitivity to HIV-1 Vpu [20], [21]. Further, they claimed that the positive selection acting on the primate Tetherin gene has been driven by ancient viral antagonists, which supports the Red Queen hypothesis [20], [21]. Here, we performed a detailed evolutionary analysis to test whether the primate Tetherin gene evolves under pervasive positive selection throughout primate evolution and examine whether the selective pressure comes from its antagonism with HIV Vpu. We obtained interesting results, in which although Tetherin as a whole experiences neutral evolution rather than pervasive positive selection throughout primate evolution, as well as in non-primate mammal evolution, it appears to undergo different episodic adaptive evolution in different primate lineages. In particular, Tetherin undergoes positive selection in a certain primate lineage (i.e. OWMs) in late primate evolution, possibly representing an intermediate stage in transition from neutral to pervasive adaptive evolution. The finding of positive selection acting on Tetherin regions that interact with viral proteins may be the result of the host–virus interaction, supporting the red-queen hypothesis.

## Results

### Phylogeny of the primate Tetherin gene sequences

**Figure 1:**
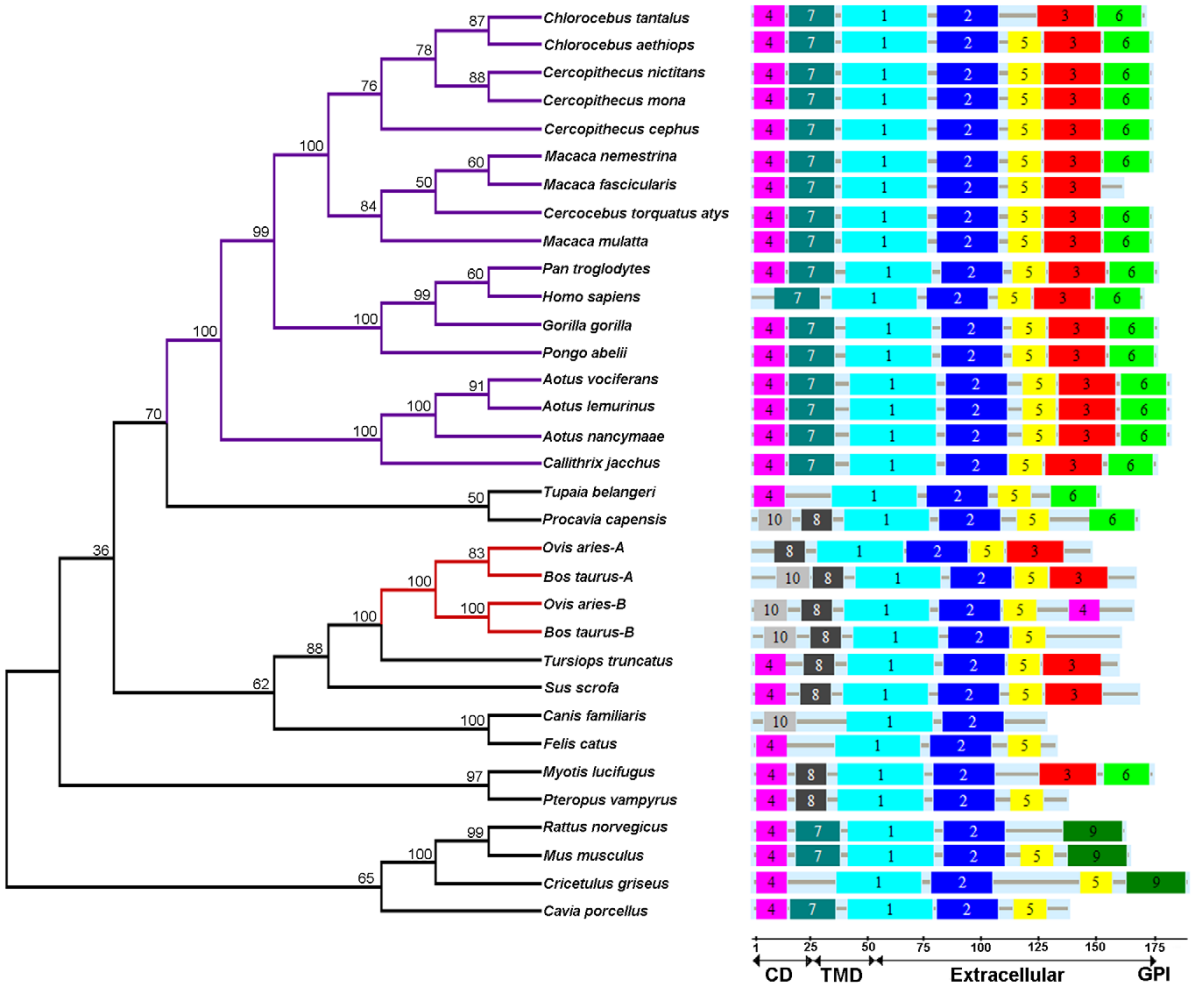
Phylogenetic tree and motif distributions of the Tetherin protein sequences from mammals. The phylogenetic tree (left panel) was constructed based on a complete alignment of 33 Tetherin protein-coding sequences using the maximum likelihood methods with 1000 bootstrap replications. Bootstrap percentages are shown at interior nodes. The primate species are shown in purple. The ruminant species are shown in red. The location of identified motifs in Tetherin is shown in the right panel. The domain maps (CD, Cytoplasmic domain; TMD, Transmembrane domain) of the Tetherin protein are predicted using SMART.

All known and predicted Tetherin gene sequences in mammals were obtained by protein databases or genome assembly searches. A maximum likelihood tree was constructed based on the protein-coding sequences of Tetherin (Figure 1, left panel). From the tree, we can clearly find that the relationships of these sequences are consistent with the known species phylogeny. Seventeen primate Tetherin sequences form a statistically supported monophyletic group (Bootstrap value  = 100%). Among the primate clade, the sequences are further divided into three statistically supported subgroups, Old World Monkeys (OWMs), Hominids and New World Monkeys (NWMs). In addition, among the non-primate mammal species, gene duplication events were confirmed occurring in ruminants before the speciation between *Bos Taurus* and *Ovis aries* [22]. Similar phylogenetic trees were also obtained by three other methods (NJ, MP and Bayesian) (Figure S1).

### Neutral evolution in primate Tetherin

**Figure 2:**
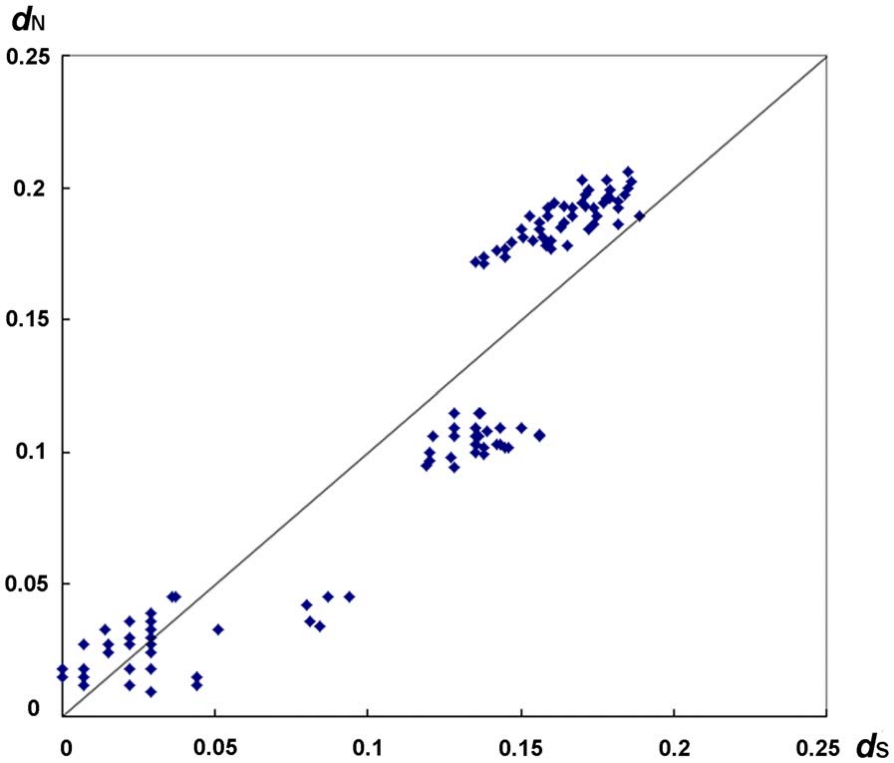
Pairwise comparisons of *d*_N_ and *d*_S_ among seventeen primate Tetherin sequences.

**Figure 3:**
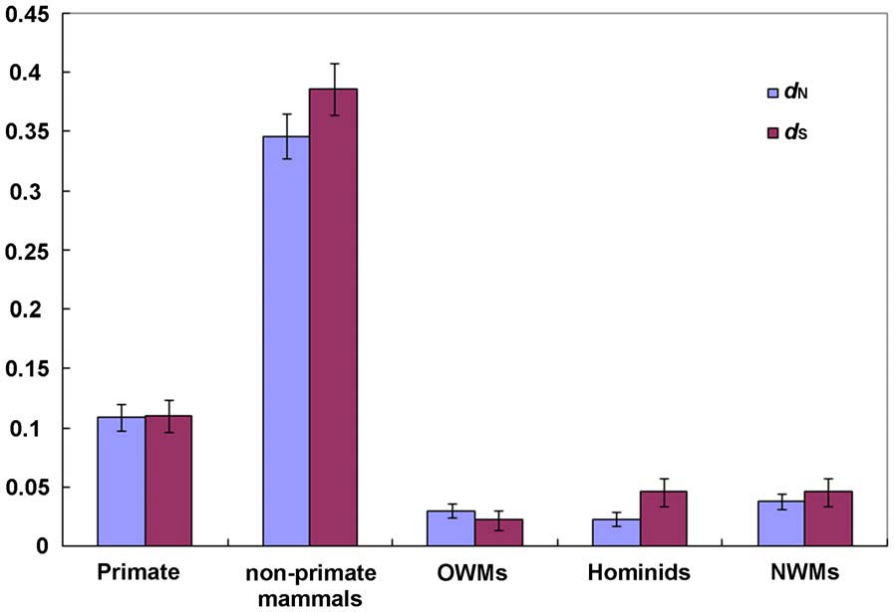
The average non-synonymous (*d*_N_) and synonymous (*d*_S_) distances in Tetherins from different groups. The transition/transversion ratios of different groups are 1.704 (primates), 0.998 (non-primate mammals), 2.391 (OWMs), 2.319 (NWMs) and 2.226 (Hominids). The error bars represent the standard errors.

The non-synonymous to synonymous rate ratio *d*_N_/*d*_S_ is an indication of the change of selective pressures. The *d*_N_/*d*_S_ ratios of <1,  = 1 and >1 indicate purifying selection, neutral evolution and positive selection on the protein involved, respectively. To investigate the evolution situation of the primate Tetherin gene, we first calculated the non-synonymous (*d*_N_) and synonymous (*d*_S_) distances between each pair of the primate Tetherin sequences (Figure 2). Intriguingly, unlike primate APOBEC3G [17], there is no significantly higher *d*_N_ than *d*_S_ in 136 pairwise comparisons of the primate Tetherin genes (p>0.05, Fisher's exact test). In particular, almost all of the points (131 of 136 pairwise comparisons) in the Figure 2 lie near the diagonal that indicates *d*_N_  =  *d*_S_ (p>0.05, Z-test), strongly suggesting neutral evolution. Further, we calculated the average *d*_N_ and *d*_S_ of the primate Tetherin sequences. The average *d*_N_ and *d*_S_ are 0.109 and 0.110, respectively (Figure 3), and the difference (*d*_N_/*d*_S_  = 0.991) between them is not significant (p>0.05, Fisher's exact test), also supporting neutral evolution. To test whether neutral evolution of Tetherin within primate species is an extraordinary exception, we compared the average *d*_N_ and *d*_S_ within non-primate mammal species. The result shows that the average *d*_N_ (0.346) is lower than *d*_S_ (0.386) for the non-primate sequences (p = 0.692, Fisher's exact test) (Figure 3), suggesting that Tetherin within non-primate mammal species also undergoes neutral evolution, and even purifying selection (*d*_N_/*d*_S_  = 0.896).

**Figure 4:**
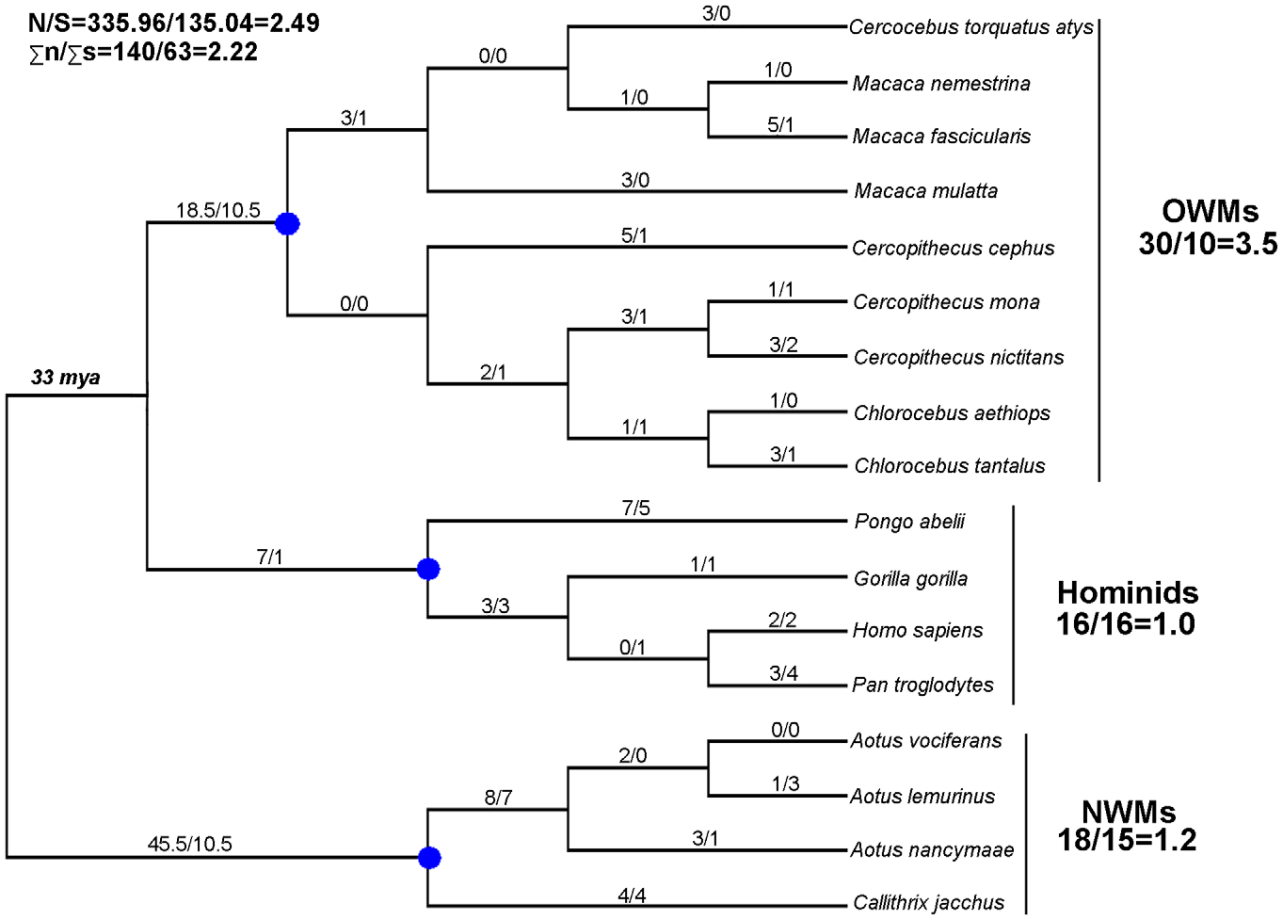
Numbers of non-synonymous (n) and synonymous (s) substitutions in the primate Tetherin. The phylogenetic tree was constructed based on a complete alignment of 17 primate Tetherin protein-coding sequences. Shown above each branch is the n/s value. N and S are the potential numbers of non-synonymous and synonymous sites, respectively. The n/s ratios of three primate lineages (excluding their ancestral branches) are shown below their names. Three blue solid nodes represent the ancestors of three primate lineages: old world monkeys (OWMs), hominids, and new world monkeys (NWMs). NWMs diverged from OWMs and hominids about 33 mya (million years ago).

Because these pairwise distances are not independent from each other, we further statistically test the hypothesis of neutral evolution using a phylogeny-based approach that compares the numbers of non-synonymous (n) and synonymous (s) on each tree branch to the potential numbers of non-synonymous (N) and synonymous (S) sites [23]. The phylogenetic relationships of the 17 primate Tetherin sequences are re-established using PHYML (Figure 4). Similar to the ML tree in Figure 1, three primate subgroups, OWMs, Hominids and NWMs, are also well classified in this ML tree. The ancestral Tetherin gene sequences at all interior nodes of the tree were inferred based on this tree using the ANC-GENE software [24]. Because the species involved are closely related, this inference exhibits high reliability with the posterior probabilities >99% for each of the ancestral sequences. Then, the numbers of n and s substitutions on each branch of the ML tree were counted using the modified Nei–Gojobori method implemented in MEGA 4.0 [25] (Figure 4). The sums of n and s for all branches are 140 and 63, respectively. The potential numbers of N and S sites are 335.96 and 135.04, respectively. The n/s ratio (2.22) is not statistically significantly different from the N/S ratio (2.49) (p = 0.580, Fisher's Exact Test), suggesting that the primate Tetherin is subject to neutral evolution as a whole during the whole evolutionary history of primate, consistent with the result of the pairwise comparison (Figure 2).

### Different selective pressures on three primate lineages

Although the primate Tetherin evolves under neutral evolution as a whole, various selective pressures were observed within three primate lineages. Within the OWM lineage, the n/s ratio (35/10 = 3.50) is 1.4 times higher than N/S  = 2.49 although not reaching statistical significance level (p = 0.231, Fisher's exact test), suggesting the action of weak positive selection on Tetherin in the OWM lineage. In contrast, the n/s ratio within the Hominid lineage, is 16/16 = 1.00 and is significantly lower than N/S  = 2.49 (p = 0.011, Fisher's exact test), strongly suggesting the action of purifying selection on Tetherin in the hominid lineage. Similarly, within the NWM lineage, the n/s ratio is 18/15 = 1.20 and is lower than N/S  = 2.49 (p = 0.036, Fisher's exact test), suggesting purifying selection on Tetherin in this lineage. On the other hand, we calculated separately the average *d*_N_ and *d*_S_ distances within three primate lineages. This result is well consistent with the above observations (Figure 3). Therefore, we conclude that Tetherin experiences different selective pressures in different primate lineages.

Although we demonstrated that Tetherin undergoes weak positive selection in the OWM lineage, and purifying selection in the hominid and NWM lineages, the selective pressures acting on the ancestral branches leading to three primate lineages remain unclear. To address this issue, the branch-site model implemented in the codeML program in the PAML package was used. This model accounts for variation in selective pressure both among sites and among lineages and is able to detect positive selection at individual sites along a specific lineage [26], [27]. We found that only when the ancestral branch of the NWM lineage was considered as the foreground branch, the branch-site model exhibits a better fit to the data (p = 0.003, χ^2^-test), and shows the signal of positive selection on the ancestral branch of NWM Tetherin (Table 1). Along this branch five sites appear to be under positive selection (ω >1 with posterior probabilities of >0.90) (Table 1). As a consequence, in the NWM lineage, Tetherin undergoes positive selection in the early stage and purifying selection in the late stage of evolution. A reverse evolutionary pattern, however, was observed in the OWM lineage, among which Tetherin undergoes neutral evolution in the early stage and weak positive selection in the late stage of evolution. This result, together with the results of n/s tests, suggests that the primate Tetherin experiences an episodic adaptive evolution.

**Table 1:** Maximum likelihood (ML) estimates for Tetherin genes.

Models	d.f.	Parameters under null model	Parameters under alternative model	InL_0_(InL_1_)	2Δℓ	*p*-value	Positively selected sites*
**Site-specific Model M7 vs. M8**							
Primates	2	p = 0.020, q = 0.011	p_0_ = 0.957, p = 0.031, q = 0.018, (p_1_ = 0.043), ω = 4.78	−1679.72 (−1683.01)	6.58	0.037 (*P*<0.05)	9C(0.965),10R(0.926), 14E (0.963), 36I (0.931)
Primates excluding *Homo sapiens*	2	p = 0.028, q = 0.016	p_0_ = 0.963, p = 0.029, q = 0.017 (p_1_ = 0.037) ω = 6.54	−1751.83 (−1760.37)	17.08	0.0002 (*P*<0.05)	9R(0.965),10K(0.926), **17W(0.963)**, 39L(0.931)
**Branch-site model A**							
OWMs group as foreground MA' vs. MA	1	**MA'** (fix ω_2_ = 1) *P*_0_ = 0.365, ω_0_ = 0,*P*_1_ = 0.635 (*P*_2a_+*P*_2b_ = 0)	**MA** *P*_0_ = 0.365, ω_0_ = 0, *P*_1_ = 0.635, ω_2_ = 1.00 (*P*_2a_+*P*_2b_ = 0)	−1682.91 (−1682.91)	0	1.000	None
Hominids group as foreground MA' vs. MA	1	**MA'** (fix ω_2_ = 1) *P*_0_ = 0.161, ω_0_ = 0, *P*_1_ = 0.263 (*P*_2a_+*P*_2b_ = 0.576)	**MA** *P*_0_ = 0.269, ω_0_ = 0, *P*_1_ = 0.436, ω_2_ = 4.05 (*P*_2a_+*P*_2b_ = 0.295)	−1682.54 (−1682.64)	0.45	0.504	None
NWMs group as foreground MA' vs. MA	1	**MA'** (fix ω_2_ = 1) *P*_0_ = 0.277, ω_0_ = 0, *P*_1_ = 0.336 (*P*_2a_+*P*_2b_ = 0.387)	**MA** *P*_0_ = 0.365, ω_0_ = 0, *P*_1_ = 0.433, ω_2_ = 7.96 (*P*_2a_+*P*_2b_ = 0.201)	−1676.22 (−1680.63)	8.82	0.003 (*P*<0.05)	17D(0.926),46I(0.944), 76E(0.948),104A(0.945), 132A(0.917)
Ruminants group as foreground MA' vs. MA	1	**MA'** (fix ω_2_ = 1) *P*_0_ = 0.160, ω_0_ = 0.195, *P*_1_ = 0.174 (*P*_2a_+*P*_2b_ = 0.667)	**MA** *P*_0_ = 0.326, ω_0_ = 0.203, *P*_1_ = 0.345, ω_2_ = 4.75 (*P*_2a_+*P*_2b_ = 0.329)	−2810.23 (−2813.48)	6.50	0.011 (*P*<0.05)	45L(0.974),51R(0.995) 71N(0.916),74L(0.963) 79N(0.995),98T(0.988) 120L(0.931), 126Q(0.966)

*The P values in parentheses are the posterior probabilities of the positively selected sites. Only the posterior probability above 0.90 was shown in the table. Codon positions from top to bottom according to the Tetherin sequence of *Homo sapiens*, *Macaca mulatta*, *Homo sapiens* and *Ovis aries*-B, respectively.

### Positive selection on the region interacting with viral protein

**Figure 5:**
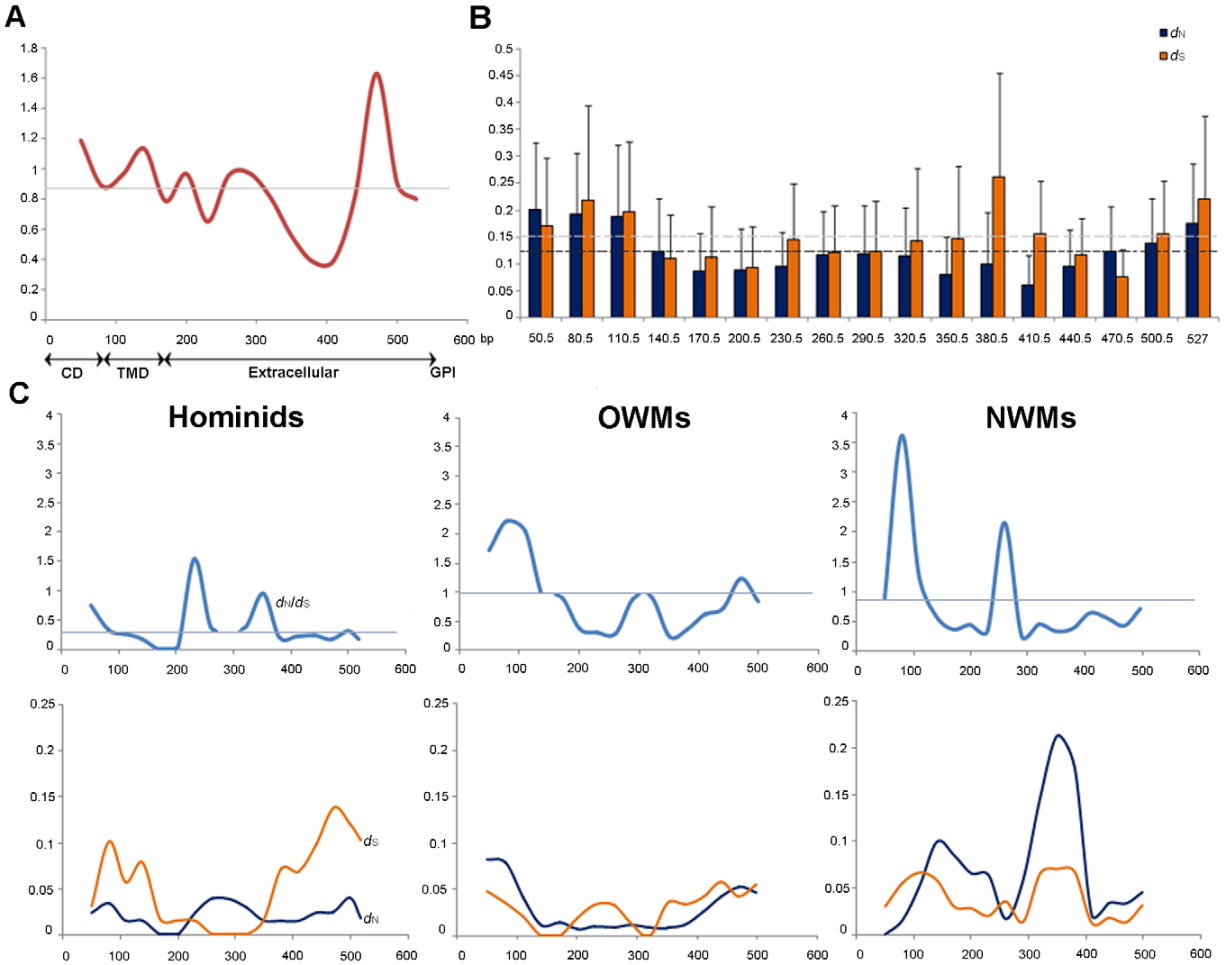
Sliding-window analyses of average *d*_N_ and *d*_S_ among the primate Tetherin sequences. **A.** Sliding window *d*_N_/*d*_S_ test among 17 primate Tetherin sequences. Numbers on the x-axis represent the sequence positions followed below by the domain map of Tetherin. **B.** Sliding-window analysis of average *d*_N_ and *d*_S_ among 17 primate Tetherin sequences. The middle position of each window on Tetherin is given on the X-axis. The bold and thin dashed lines show the average *d*_N_ and *d*_S_ for the entire sequences, respectively. The error bar shows one standard error. **C.** Upper panel: Sliding window *d*_N_/*d*_S_ tests on three primate lineages (Hominids, OWMs and NWMs). The *d*_N_/*d*_S_ value is not shown for part of the curve because *d*_S_ is zero in this region (see plot below). Lower panel: Sliding-window analyses of average *d*_N_ and *d*_S_ in three primate lineages. The sliding-window analyses were performed using a 100-bp sliding window moving in steps of 30-bp. The straight lines shown in **A** and **C** represent the average *d*_N_/*d*_S_ ratios.

Positive selection usually affects small regions of gene involved. However, the whole-gene analysis is notoriously poor at detecting specific domains under positive selection, especially when the rest of a gene is subject to purifying selection [28]. To investigate which region of the primate Tetherin gene is under positive selection, we performed a sliding window *d*_N_/*d*_S_ test [29]. We first performed a sliding window (100-bp window; 30-bp slide) analysis on all primate Tetherin sequences. The result shows that some small regions located in cytoplasm, transmembrane, and ectodomain have been under positive selection (Figure 5 A and B), well consistent with the previous reports [20], [21]. Then, we performed the sliding window *d*_N_/*d*_S_ tests on three primate lineages and found different results in different primate lineages (Figure 5C). A similar *d*_N_/*d*_S_ ratio curve to that of all primate Tetherin sequences was observed in the OWMs (Figure 5C). Both the N- and C-terminal regions of the OWM Tetherin have *d*_N_/*d*_S_ ratios of >1. In the NWMs, regions located in N-terminal and ectodomain have been under positive selection (Figure 5C). In the hominids, a region in ectodomain appears to be under positive selection. In addition, although the N-terminal region of the hominid Tetherin has the *d*_N_/*d*_S_ ratio of 0.76, it is significantly higher than the average of *d*_N_/*d*_S_ ratio (0.39) over the entire hominid Tetherin gene (P<0.01, Z-test), possibly implying a relaxation of selective constraint on this region.

In addition to the sliding-window analyses, we further detected positive selection on the primate Tetherin using the site model in PAML package. There are only four sites to be detected under positive selection (with posterior probabilities of >0.90) (Table 1), less than that of previous studies [20], [21], possible due to the usage of different method and different sequences. However, when we used the random effects likelihood (REL) program (HyPhy) to detect positive selection, a similar result to the previous reports was obtained (Table S1). From these results, we noted that most of these sites focus on the regions that interact with viral proteins.

Since *Homo sapiens* Tetherin lacks a five-amino-acid motif interacting with SIV Nef [30], given that this motif undergoes positive selection, it will be another compelling evidence for the host–virus arms race. To address this issue, we removed *Homo sapiens* Tetherin sequence from the analyzed sequence data. As expected, a residue at site 17 located in this motif was detected under significant positive selection (with a posterior probability of >0.95) (Table 1 and Table S2), indicating that the SIV Nef-interacting motif is indeed under positive selection. Importantly, the residue at site 17 was recently experimentally confirmed to determine susceptibility of Tetherin to Nef antagonism [31].

### No bias in amino acid changes

An amino acid substitution can be classified as either conservative or radical, depending on whether it leads to a change in a certain physicochemical property of the amino acid. In many proteins, the amino acid substitutions caused by positive selection are non-random [32], [33]. For instance, in primate APOBEC3G evolution, positive selection favors alterations of amino acid charge, which involves the interaction of APOBEC3G and HIV-1 Vif [17]. To investigate whether this is the case in primate Tetherin, especially in the OWM Tetherin that undergoes weak positive selection, we estimated radical and conservative non-synonymous (n) substitutions on each branch of the tree (Figure S2). The radical n substitutions are defined as those that can alter the charge, polarity, and size & polarity of the encoded amino acids that are very important for the structure and the function of a protein, whereas the conservative n substitutions do not alter them [32]. We found that the radical n substitution rate (r/R) is slightly lower than the conservative n substitution rate (c/C) in the primate Tetherin gene (Table 2). Different result was observed in the OWM Tetherin in which the radical n substitution rate (r'/R) appears to be slightly higher than the conservative n substitution rate (c'/C) in charge and size ` polarity (p = 0.298 and 0.364, respectively, Fisher's Exact Test) (Table 2). These findings suggest that there may be no obvious bias in the alterations of amino acids during the evolution of the primate Tetherin, and imply that the primate Tetherin may retain broad spectrum of antiviral activity by maintaining structure stability.

**Table 2:** Numbers of conservative and radical non-synonymous substitutions on the branches.

	R^a^	C^a^	∑r^b^	∑c^b^	r/R^c^	c/C^c^	r'/R^d^	c'/C^d^
**Charge**	133.15	202.81	51.50	88.50	0.39	0.44	0.120	0.094
**Polarity**	102.48	233.48	40.00	100.00	0.39	0.42	0.078	0.116
**Size & polarity**	216.63	119.33	86.50	53.50	0.40	0.45	0.108	0.096

a The potential numbers of radical non-synonymous substitutions and conservative non-synonymous substitutions.

b The total numbers of radical non-synonymous substitutions and conservative non-synonymous substitutions on all branches.

c The total radical and conservative non-synonymous substitution ratios of all branches.

d The total radical and conservative non-synonymous substitution ratios of OWMs.

### Accelerated evolution after Tetherin gene duplication in ruminants

Since gene duplication of Tetherin was detected in ruminants (Figure 1) and duplicated Tetherins *Ovis aries*-A and –B were recently demonstrated to have different antiviral activity [22], we further tested whether the duplicated genes undergo accelerated evolution. When the ruminant lineage was considered as the foreground branch, the branch-site model exhibits a better fit to the data (p = 0.011, χ^2^-test), and shows that positive selection is the driving force of this unique duplication event (Table 1). Meanwhile, 6 sites were detected under significant positive selection with posterior probabilities of >0.95 (Table 1). These results suggest that duplicated Tetherin genes undergo an accelerated evolution process and the positively selected sites may contribute to the significant difference in the antiviral activity between *Ovis aries*-A and -B Tetherins.

### Conserved Domains and/or Motifs in Tetherin

**Figure 6:**
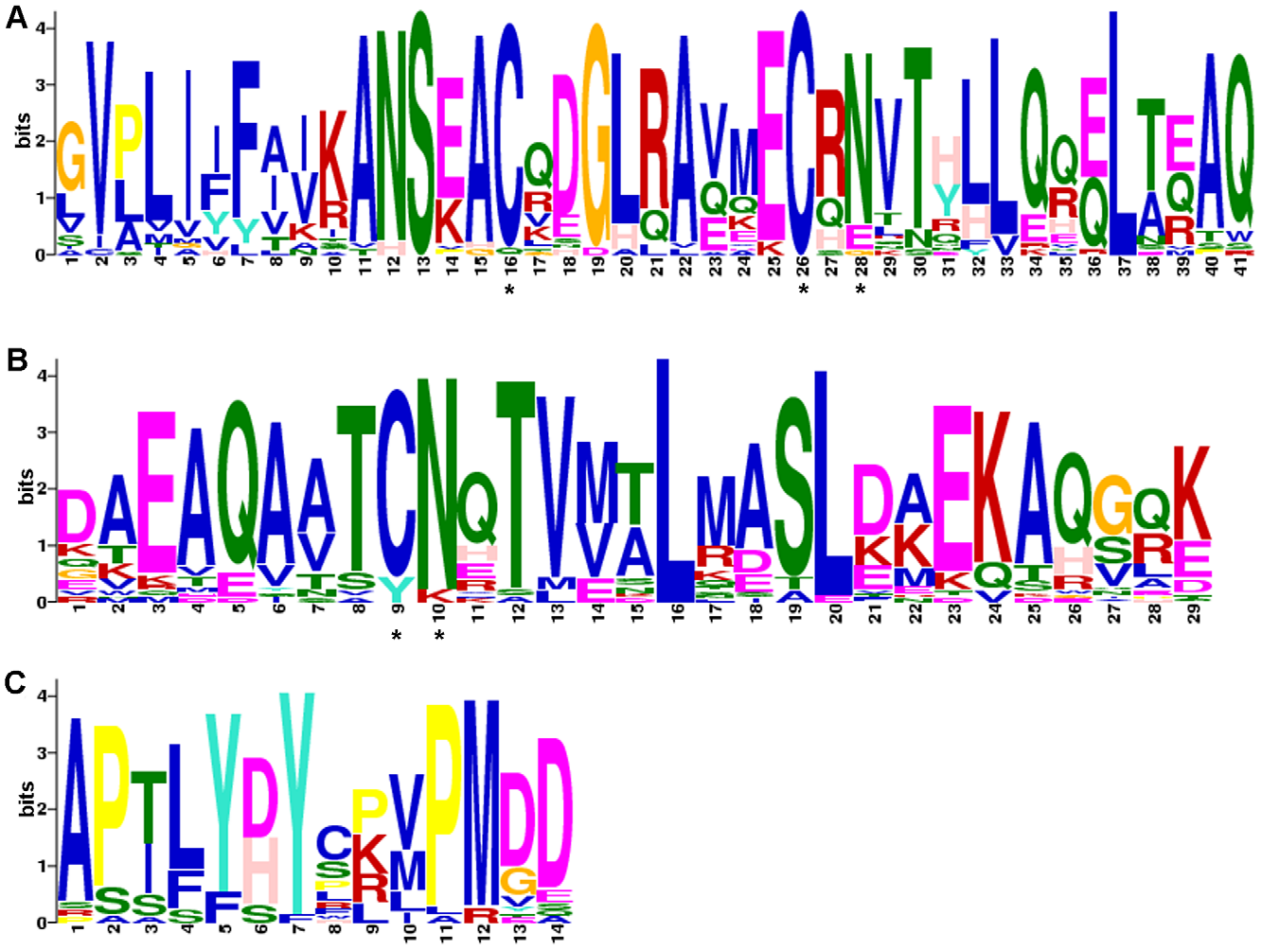
Sequence logos (MEME LOGOs) of conserved motifs identified in Tetherin. **A**. Sequence logos of motif 1. **B**. Sequence logos of motif 2. **C.** Sequence logos of motif 4. The character and size of each logo represent the proportion of an amino acid at the specific site. The YxY motif associated with the clathrin-mediated endocytosis is located in the positions 5–7 in motif 4. Two asparagines (N) and three cysteines (C) that are responsible for the glycosylation and dimerization of Tetherin, respectively, are highlighted by asterisks in motifs 1 and 2. For detail, see the right panel of Figure 1 and Figure S3.

We further investigated the domain and/or motif distribution of Tetherin. The Tetherin amino acid sequences were firstly subjected to a search to find matching Pfam families, and no significant match was found in the Pfam database. We then performed motif analysis using the MEME/MAST software. The results show that all Tetherin sequences (including those from non-primate mammals) contain two highly conserved motifs 1 and 2 located in the extracellular region (Figure 1, right panel), implying that the two motifs are functional important for Tetherin. In motifs 1 and 2, two asparagines (N) and three cysteines (C) are attractive due to that they are responsible for the glycosylation and dimerization of Tetherin, respectively (Figure 6 A and B, and Figure S3) [14]. The presence of these conserved sites in the primate Tetherin, as well as in the mammalian orthologs, may suggest that the non-primate mammal Tetherin exerts similar biological activity as the primate Tetherin. In addition, we found that motif 4 located in the N-terminal contains an YxY motif that is associated with the clathrin-mediated endocytosis (Figure 6C) [34]. Although not completely conserved in Tetherins, all YxY motifs contain at least one tyrosine, implying that the presence of one tyrosine in this motif is enough for Tetherin to participate in the clathrin-mediated endocytosis [34]. Intriguingly, four ruminant Tetherins do not contain the YxY motif, suggesting that these Tetherins may have another pathway for cellular trafficking.

## Discussion

Tetherin is a novel antiviral restriction factor that is antagonized by HIV Vpu in a species-specific manner [8]. It is reminiscent of the APOBEC3G and TRIM5a, another two well-known antiviral restriction factors, which counteract HIV. The APOBEC3G and TRIM5a have been demonstrated to evolve under pervasive positive selection throughout primate evolution, well supporting the red-queen hypothesis [17], [18], [19], [35], [36]. Therefore, one naturally presumes that the Tetherin also evolves under pervasive positive selection throughout primate evolution, especially when few amino acid sites in Tetherin have been identified under positive selection [20], [21]. Here, although we detected some positively selected sites in the primate Tetherin, we found that Tetherin as a whole experiences neutral evolution rather than pervasive positive selection throughout primate evolution, as well as in non-primate mammal evolution. In particular, Tetherin appears to undergo different episodic adaptive evolution in different primate lineages.

We found that the primate Tetherin has been under positive selection as early as 33 million years ago when NWMs diverged from OWMs and hominids [37], suggesting that the selective pressure exerting on the primate Tetherin in the early stage may come from ancestral viruses. However, these ancestral viruses are less likely to be lentiviruses, because the common ancestor (gray mouse lemur prosimian immunodeficiency virus, pSIVgml) of the primate lentivirus family is estimated to be around 4.2–14 million years old [38], [39]. In addition, only the OWM group was detected to be under weak positive selection in the late stage (Figure 4). This result may be attributed to different situation of viral infections among three subgroups. In OWMs, lentiviral infection can be traced back to a much longer time ago compared with that in hominids [40]. Moreover, unlike the lentiviruses that can infect OWMs and hominids, and result in ongoing host–virus arms race, the infection by non-lentiviruses is often an occasional zoonosis [41], [42], and is unlikely to become an ongoing selective force, providing a possible explanation why NWM Tetherin does not undergo a continuous adaptive evolution.

Although the primate Tetherin undergoes different episodic adaptive evolution in different lineages, the regions of Tetherin, especially the N-terminal and transmembrane regions, which interact with viral proteins, are under positive selection (in OWMs and NWMs) or relaxed purifying selection (in hominids). Moreover, the positively selected sites identified in the primate Tetherin generally focus on these regions. These results, together with the recent studies that demonstrated that some of the positively selected sites in the regions interacting with Vpu (HIV-1) and Nef (SIV) are able to determine susceptibility of Tetherin to viral proteins, strongly indicate that the selective pressure acting on the N-terminal and transmembrane regions of Tetherin comes from viruses, especially the lentiviruses. Intriguingly, we also found some regions located in the ectodomain under positive selection. These regions may be also related to the interaction between Tetherin and viral proteins and should be seriously considered in future study.

Nef (SIV) counteracts most primate Tetherins but not human Tetherin because human Tetherin lacks a 5-amino-acid motif that interacts with Nef. After removing human sequence from the analysis, the Nef-interacting region (especially site 17) is under obvious positive selection, further supporting the above observation that the selective pressure on the primate Tetherin comes from viruses. On the other hand, instead of Nef, HIV-1 evolves its protein Vpu to antagonize human Tetherin [31], [43], [44], implying an ongoing host-virus arms race.

Unlike APOBEC3G and TRIM5a that evolve under pervasive positive selection throughout primate evolution, and CD209 (DC-SIGN) and TRIM19 that exhibit additional important physiological functions in primates apart from interaction with viruses and experience purifying selection throughout primate evolution [45], [46], the primate Tetherin as a whole experiences neutral evolution and undergoes positive selection in a certain primate lineage (i.e. OWMs) in late primate evolution, possibly representing an intermediate stage in transition from neutral to pervasive adaptive evolution. However, why does the primate Tetherin experience this extraordinary evolutionary history?

First, the antiviral activity of Tetherin is apparently broad and nonspecific. Since Tetherin broadly restricts the release of enveloped viruses from the surface of infected cells by physically linking viral and cellular membranes [43], it needs to maintain a certain degree of stability. Moreover, quite distinct from the primate APOBEC3G [10], there is no obvious bias in the alterations of amino acids in the evolution of the primate Tetherin, also suggesting that Tetherin retains its broad spectrum of antiviral activity by maintaining structure stability. Second, for Tetherin to work, it has to maintain interactions with conserved elements of the cellular trafficking and endocytosis machinery. Third, the artificial Tetherin that lacks conserved features of native Tetherin (e.g. the glycosylation sites in the ectodomain) was recently found to retain full antiviral activity, suggesting that the overall configuration rather than partial specific sequence of Tetherin is important for antiviral activity [10]. It implies that in addition to its antiviral activity, Tetherin might have other important physiological functions. The detection of strongly purifying selection on some regions of the ectodomain supports this point (Figure 5). In addition, although the complete role of Tetherin in mammals remains unclear, limited data show that Tetherin can (at least) mediate a specific feedback mechanism to turn off interferon production by plasmacytoid dendritic cells [8], [47]. The role in negative feedback for interferon production might be a potential reason of evolutionary conservation of some regions in the primate Tetherin gene. Finally, the primate lentiviruses have an obvious shorter history than primates, and the ongoing primate (i.e. OWMs and hominids) lentiviruses interaction (coevolution) is a relatively recent event, which possibly explains the positive selection on Tetherin in OWMs and relaxed purifying selection in hominids.

Gene duplication enables a genome or species to enhance its capacity in adapting to changing environments. Apart from the extraordinary evolutionary history of the primate Tetherin, the phylogenetic tree confirmed gene duplication previously reported in ruminant Tetherins [22]. The duplicated Tetherins have different antiviral activities. For example, *Ovis aries*-A Tetherin exhibits higher antiviral activity than *Ovis aries*-B Tetherin [22]. The branch-site model analysis shows that positive selection accelerates Tetherin evolution in ruminant lineage. Six positively selected sites identified in these duplicate Tetherins may contribute to their obvious difference in the antiviral activities. It is well known that APOBEC3 and TRIM gene families expand in primates in response to virus infection [17], [48], [49]. Therefore, the change in antiviral activities between duplicated Tetherins in ruminants may be attributed to some viral infections, and the duplicated copies with increasing antiviral ability may serve as host strategy to counter virus infection. However, no gene duplication was found in the primate Tetherin genes. Gene duplication event is difficult to happen when genes undergo neutral evolution. If a duplicated gene is selectively neutral, it only has a small probability of being fixed and most duplicated genes will be lost [50]. Moreover, the fixation of duplicated genes is very time consuming and needs some selection pressures. As mentioned above, the primate Tetherin as a whole experiences neutral evolution. In addition, weak positive selection occurring in the OWM Tetherin and relaxed selective constraint in the hominid Tetherin may just represent an initiation of accelerated evolution of the primate Tetherin. Therefore, no gene duplication occurring in the primate Tetherin may be due to a relative short history of accelerated evolution. Given that the evolution of the primate Tetherin is continuously accelerated due to the ongoing lentiviral infection, the occurrence of gene duplication in the primate Tetherin may be just a matter of time.

Since the Tetherin sequences of many other species have not been identified, our study should not be regarded as the most comprehensive study on the molecular evolution of Tetherin. So the concern on the complex evolutionary history of the Tetherin in mammals needs to be continued. Moreover, the intriguing questions about the origin of Tetherin and its complete role in mammals also deserve to be considered.

## Materials and Methods

### Sequence Data Collection

The rodent and the primate Tetherin gene sequences reported previously were retrieved from the National Center for Biotechnology Information (NCBI). PSI-BLAST and TBLASTN searches were performed in protein databases or genome assemblies at NCBI, ENSEMBL, the Sanger Institute, and UCSC Genome Bioinformatics Group using these known Tetherin protein sequences. All searches had been performed in several iterations using default parameters. In addition, an HMM (Hidden Markov Model) search was carried out in the protein database at UniProt using the HMMER 2.3.2 software package [51]. Each newly identified putative Tetherin sequence was used as a query using BLAST against the non-redundant GenBank database to check whether their best hit was a Tetherin gene. Constructed open reading frames (ORFs) were conceptually translated into amino acid and checked against their closest homologs.

After removing the redundant sequences and a predicted horse Tetherin sequence that is too short to analyze, 17 primate and 16 non-primate mammal Tetherin sequences were used in this study. The 17 primate sequences include *Chlorocebus tantalus* (FJ345303), *Macaca nemestrina* (DY743778), *Macaca mulatta* (GQ304749), *Cercocebus torquatus atys* (FJ864714), *Pan troglodytes* (XM_512491), *Homo sapiens* (NM_004335), *Aotus vociferans* (FJ638418), *Chlorocebus aethiops* (FJ943430), *Aotus nancymaae* (FJ638415), *Macaca fascicularis* (CJ479048), *Gorilla gorilla* (GQ925926), *Aotus lemurinus* (FJ638414), *Callithrix jacchus* (ENSCJAG00000009764), *Cercopithecus mona* (GQ925924), *Cercopithecus nictitans* (GQ925923), *Pongo abelii* (FJ626246) and *Cercopithecus cephus* (GQ864267). The 16 non-primate mammal sequences include *Felis catus* (ENSFCAT00000001009), *Bos taurus*-A (XM_871059), *Bos taurus*-B (XM_584000), *Canis familiaris* (ENSCAFG00000023046), *Tursiops truncatus* (ENSTTRG00000006805), *Procavia capensis* (ENSPCAG00000002224), *Cavia porcellus* (ENSCPOG00000010448), *Pteropus vampyrus* (ENSPVAG00000007879), *Sus scrofa* (FJ527910), *Myotis lucifugus* (ENSMLUG00000017408), *Tupaia belangeri* (ENSTBEG00000013950), *Mus musculus* (NM_198095), *Rattus norvegicus* (NM_198134), *Ovis aries*-A (GU376752), *Ovis aries*-B (GU376751) and *Cricetulus griseus* (AY272060).

### Evolutionary analyses of Tetherin sequences

The protein-coding sequences of Tetherin were aligned using CLUSTAL W program implemented in MEGA 4.0 [25] or webPRANK (http://www.ebi.ac.uk/goldman-srv/webPRANK/) [52], and then manually edited. The phylogenetic tree of all Tetherin protein-coding sequences was constructed with MP and NJ algorithms implemented in PAUP* v4.0b10 [53], as well as with ML and Bayesian algorithms using the programs PHYML v2.4.4 [54] and MrBayes v3.1.2 [55], [56], respectively. Further, the phylogenetic tree of the 17 primate Tetherin protein-coding sequences was re-established using PHYML [54]. For MP analysis, all characters were treated as unordered and equally weighted throughout. A heuristic search was performed with the maximum number of trees set to 100. For NJ, ML and Bayesian reconstructions, the optimal nucleotide substitution model (HKY+G) was chosen using Akaike information criterion (AIC) implemented in jModelTest 0.1 [57]. Relative support of internal node was performed by bootstrap analyses with 1000 replications for MP, NJ and ML reconstructions. For Bayesian reconstruction, the dataset was partitioned into codon positions and four Markov Chain Monte Carlo (MCMC) chains were used with the default temperature of 0.1. Four repetitions were run for 10,000,000 generations with tree and parameter sampling occurring every 10,000 generations. The first 25% of samples were discarded as burnin, leaving 750 trees per run. Posterior probabilities for internal node were calculated from the posterior density of trees.

The numbers of non-synonymous substitutions per non-synonymous site (*d*_N_) and that of synonymous nucleotide substitutions per synonymous site (*d*_S_) were computed using the modified Nei–Gojobori method in MEGA 4.0 [25] with consideration of transition/transversion ratios in the legend of Figure 3. The significance of difference between *d*_N_ and *d*_S_ was estimated with the Z statistics, with standard errors based on 1000 bootstrap replicates using MEGA 4.0 [25]. The ancestral Tetherin sequences at all interior nodes of the primate tree were inferred based on the phylogeny of the 17 primate species using the ANC-GENE software [24], and then the numbers of synonymous (s) and non-synonymous (n) substitutions for each branch were calculated. Sliding window analysis was performed on the primate Tetherin genes using K-Estimator software package [29]. The radical and conservative non-synonymous substitutions with regard to amino acid charge, polarity, and size & polarity were estimated using HON-NEW software [32].

The branch-site model and site-specific model of the likelihood method were performed using the program codeML implemented in PAML 4.2 software package [58] for testing positive selection on individual sites along a specific lineage and at different sites, respectively. The significance of difference between the null model and the alternative model was evaluated by calculating twice the log-likelihood difference following a χ^2^ distribution, with the number of degrees of freedom. In the branch-site model, the lineages of interest are set to be foreground, and the other lineages to be background. In branch-site model A, 3 ω ratios are assumed for foreground (0< ω_0_ <1, ω_1_ = 1, ω_2_>1) and 2 ω ratios for background (0< ω_0_<1, ω_1_ = 1). The null model (model A') is the same as model A, but ω_2_ = 1 is fixed. In the site-specific model that allows for variable selection patterns among amino acid sites, we constructed likelihood ratio tests (LRT) to compare M7 with M8. The M8 model allows for positively selected sites. When the M8 model fitted the data significantly better than the corresponding null model (M7), the presence of sites with ω >1 is suggested. The posterior probability for each codon site of being under positive selection was calculated by the conservative Empirical Bayes approach [59].

We also analyzed our datasets using HYPHY package available through the Datamonkey facility (http://www.datamonkey.org) [60]. Datamonkey includes three methods for detecting sites under selection: single likelihood ancestor counting (SLAC), fixed effects likelihood (FEL) and random effects likelihood (REL). The REL method is often the only method that can infer selection from small or low divergence alignments and tends to be the most powerful of the three test statistics. So this method was run using the HKY85 substitution model (best model chosen using AIC) on a neighbor-joining phylogenetic tree by the Datamonkey web server.

### Protein domain and Motif analyses

In order to investigate protein motifs in detail, the Tetherin protein sequences were analyzed using the MEME/MAST software (http://meme.sdsc.edu/meme/website/intro.html) [61], [62] with maximum 10 number of motifs to find. Domain analyses of Tetherin proteins were performed in Pfam domains database (http://pfam.sanger.ac.uk). The secondary structure of Tetherin protein sequences was predicted using SMART (http://smart.embl-heidelberg.de/).

## Supporting Information

Figure S1 Consensus phylogenetic tree of Tetherin protein-coding sequences by three methods (NJ, MP and Bayesian). Bootstrap percentages and Posterior probabilities obtained by the three methods (followed the order of NJ, MP and Bayesian methods) are labeled on the main branches. The symbol * means that the branch is not supported by the corresponding method.
(0.29 MB TIF)

Figure S2 Numbers of conservative non-synonymous (c) and radical non-synonymous (r) substitutions on the primate Tetherin. Conservative non-synonymous substitutions do not alter the physicochemical property of the encoded amino acid, whereas radical non-synonymous substitutions do. The r/c is labeled on the main branches for the three physicochemical properties (followed the order of charge, polarity and size and polarity).
(0.34 MB TIF)

Figure S3 The regular-expression of 10 motifs of all Tetherin protein sequences.
(1.10 MB TIF)

Table S1 Random effects likelihood (REL) result for seventeen primate Tetherin protein-coding sequences.
(0.03 MB DOC)

Table S2 Random effects likelihood (REL) result for non-human primate Tetherin protein-coding sequences.
(0.03 MB DOC)
