# Supplementary figures and images for: Molecular Evolution of the Primate Antiviral Restriction Factor Tetherin
Source: PLoS One. 2010 Jul 30;5(7):e11904. doi: 10.1371/journal.pone.0011904 (PMC2912774; doi:10.1371/journal.pone.0011904)

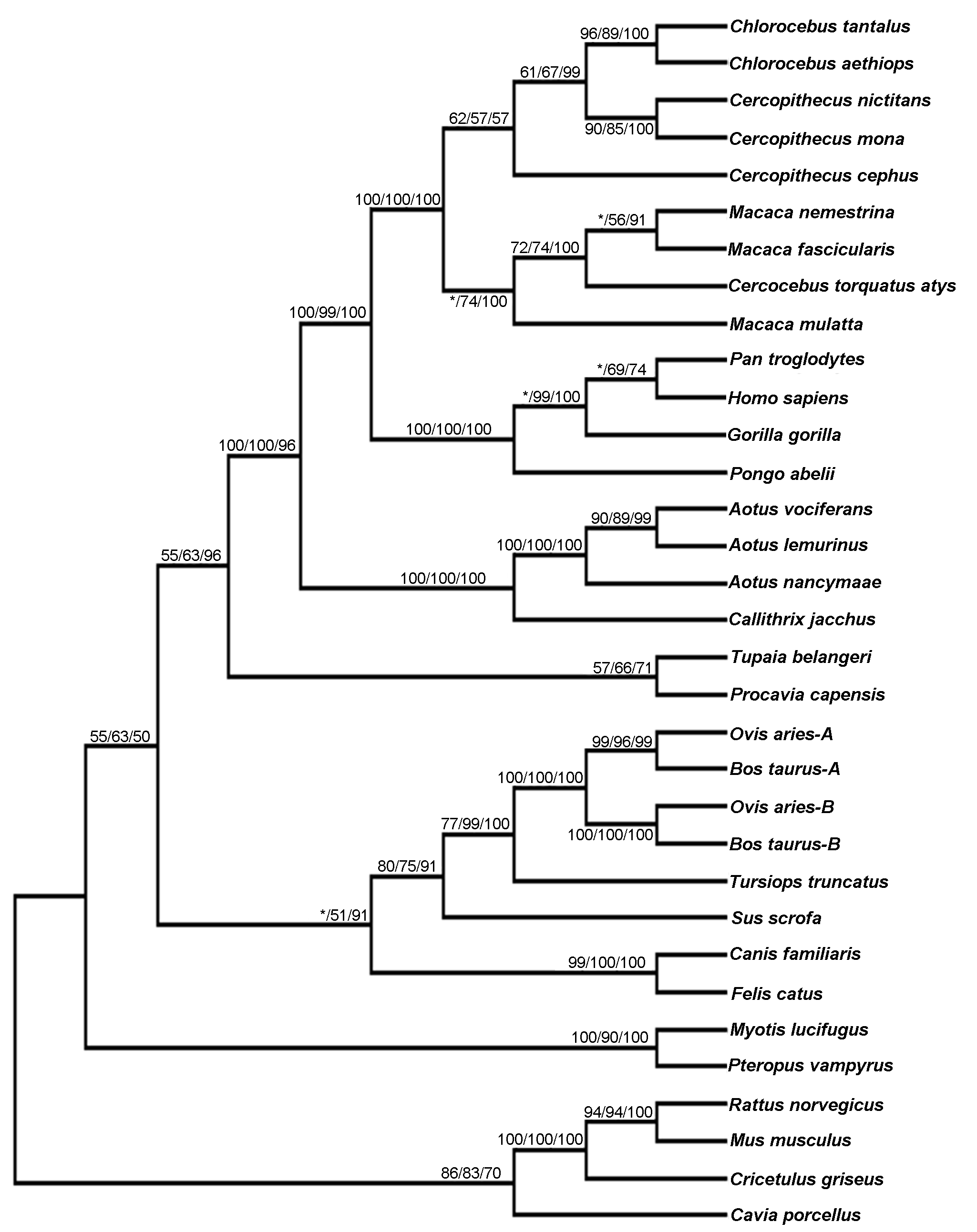

Supplement: Figure S1 — Consensus phylogenetic tree of Tetherin protein-coding sequences by three methods (NJ, MP and Bayesian). Bootstrap percentages and Posterior probabilities obtained by the three methods (followed the order of NJ, MP and Bayesian methods) are labeled on the main branches. The symbol * means that the branch is not supported by the corresponding method. (0.29 MB TIF) [file pone.0011904.s001.tif]

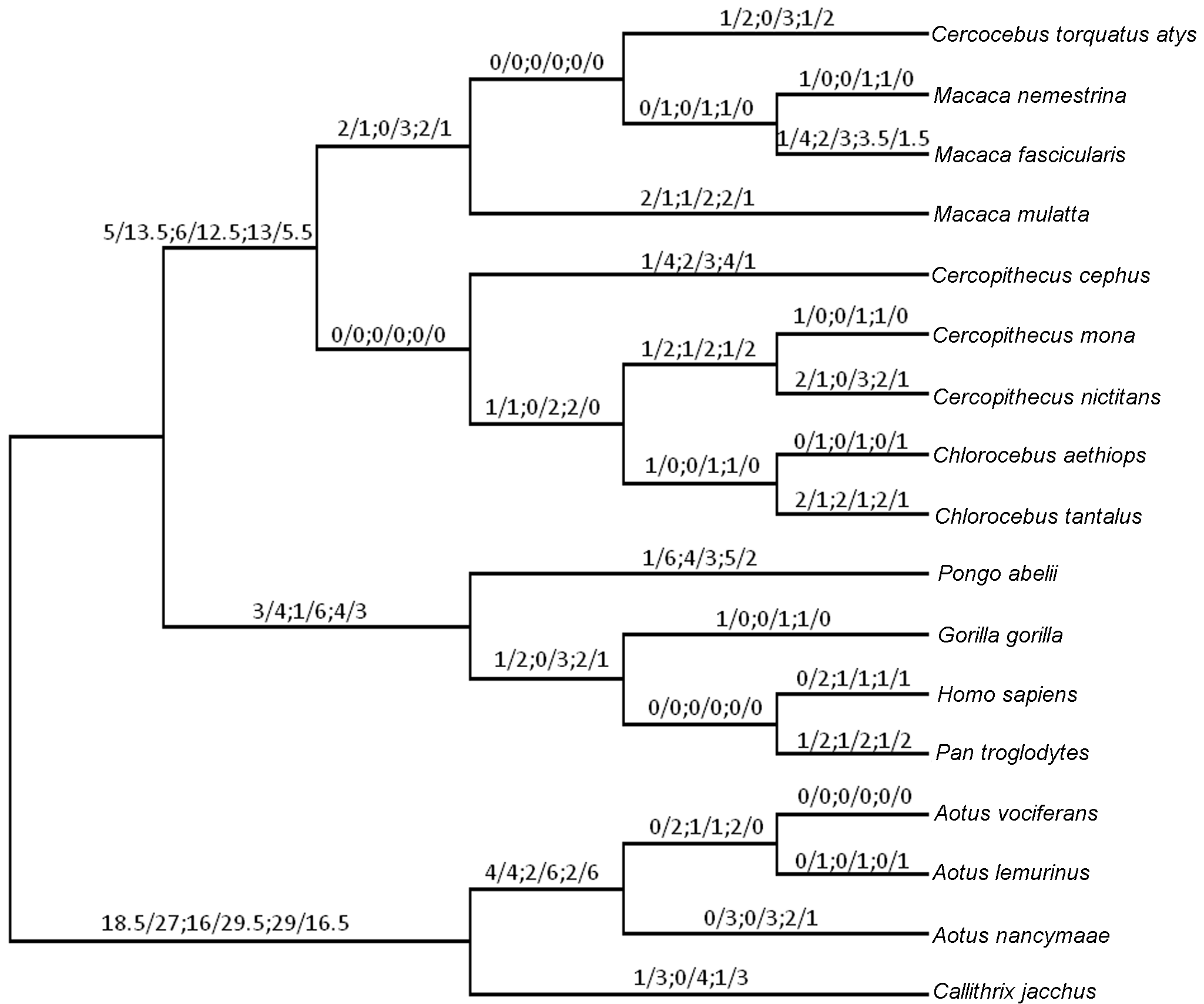

Supplement: Figure S2 — Numbers of conservative non-synonymous (c) and radical non-synonymous (r) substitutions on the primate Tetherin. Conservative non-synonymous substitutions do not alter the physicochemical property of the encoded amino acid, whereas radical non-synonymous substitutions do. The r/c is labeled on the main branches for the three physicochemical properties (followed the order of charge, polarity and size and polarity). (0.34 MB TIF) [file pone.0011904.s002.tif]

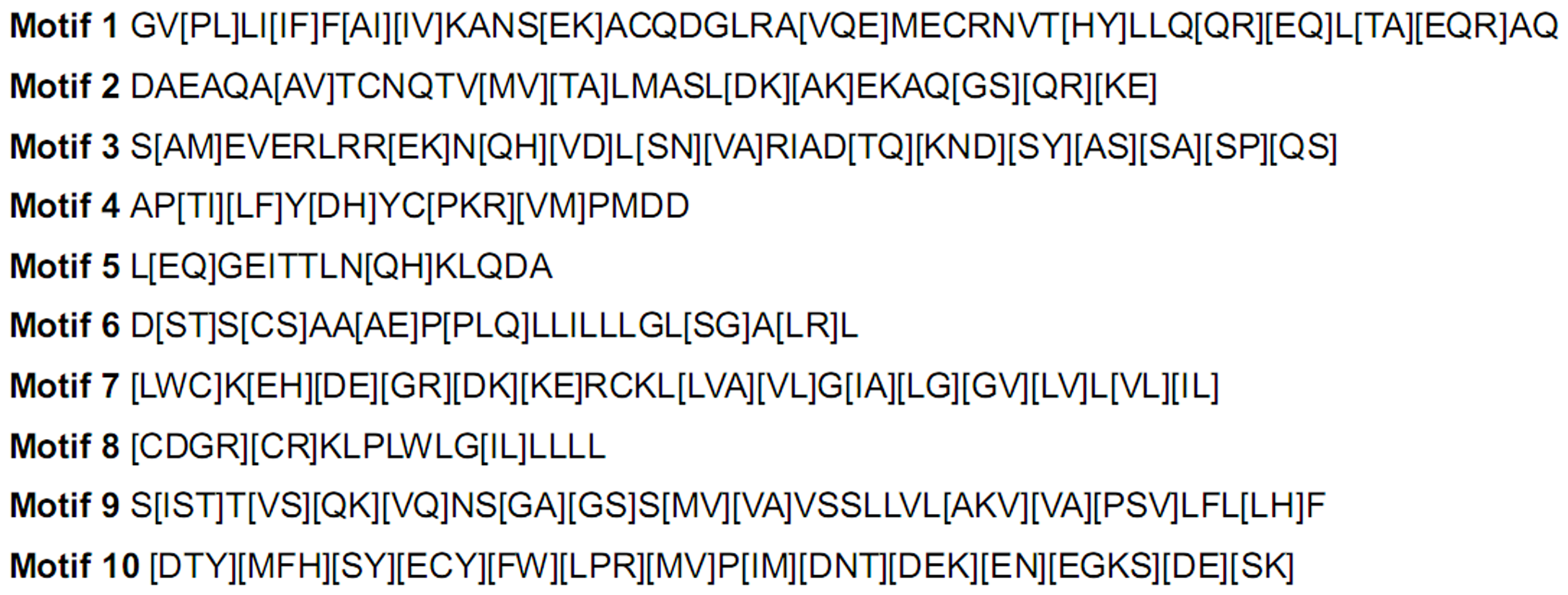

Supplement: Figure S3 — The regular-expression of 10 motifs of all Tetherin protein sequences. (1.10 MB TIF) [file pone.0011904.s003.tif]
