# Supplementary material for: Molecular Evolution of the Primate Antiviral Restriction Factor Tetherin
Source: PLoS One. 2010 Jul 30;5(7):e11904. doi: 10.1371/journal.pone.0011904 (PMC2912774; doi:10.1371/journal.pone.0011904)
Supplement: Table S1 — Random effects likelihood (REL) result for seventeen primate Tetherin protein-coding sequences. (0.03 MB DOC) [file pone.0011904.s004.doc]

**Table S1. Random effects likelihood (REL) result for seventeen primate Tetherin protein-coding sequences.**

| **Codon** | **Mean *d*_S_** | **Mean *d*_N_** | **P(*d*_N_>*d*_S_)*** | **Bayes Factor** |
| --- | --- | --- | --- | --- |
| 9 | 0.759 | 1.346 | 0.991 | 114.84 |
| 10 | 0.699 | 1.228 | 0.998 | 527.46 |
| 14 | 0.741 | 1.407 | 0.993 | 158.76 |
| 19 | 0.723 | 1.180 | 0.991 | 121.28 |
| 21 | 0.725 | 1.202 | 0.995 | 213.36 |
| 43 | 0.700 | 1.327 | 0.999 | 802.59 |
| 47 | 0.705 | 1.224 | 0.995 | 239.64 |
| 120 | 0.735 | 1.252 | 0.991 | 121.04 |
| 161 | 0.742 | 1.188 | 0.989 | 96.91 |
| 167 | 0.705 | 1.192 | 0.995 | 208.17 |
| 187 | 0.768 | 1.182 | 0.985 | 71.10 |

A Bayes factor of greater than 50 at a given site was considered to be strong support for positive selection.

*Posterior probability for positive selection (*d*_N_>*d*_S_) at the site.
