# Supplementary material for: Molecular Evolution of the Primate Antiviral Restriction Factor Tetherin
Source: PLoS One. 2010 Jul 30;5(7):e11904. doi: 10.1371/journal.pone.0011904 (PMC2912774; doi:10.1371/journal.pone.0011904)
Supplement: Table S2 — Random effects likelihood (REL) result for non-human primate Tetherin protein-coding sequences. (0.03 MB DOC) [file pone.0011904.s005.doc]

**Table S2. Random effects likelihood (REL) result for non-human primate Tetherin protein-coding sequences.**

| **Codon*** | **Mean *d*_S_** | **Mean *d*_N_** | **P(*d*_N_>*d*_S_)**** | **Bayes Factor** |
| --- | --- | --- | --- | --- |
| 9 | 0.775 | 1.442 | 0.984 | 63.60 |
| 10 | 0.699 | 1.290 | 0.996 | 246.24 |
| 14 | 0.756 | 1.282 | 0.986 | 75.94 |
| **17** | 1.951 | 7.164 | 0.982 | 58.74 |
| 19 | 0.732 | 1.233 | 0.988 | 86.05 |
| 21 | 0.737 | 1.256 | 0.990 | 101.01 |
| 43 | 0.701 | 1.416 | 0.996 | 272.08 |
| 47 | 0.708 | 1.284 | 0.993 | 156.36 |
| 120 | 0.771 | 1.322 | 0.982 | 55.68 |
| 161 | 0.753 | 1.249 | 0.985 | 68.06 |
| 167 | 0.708 | 1.247 | 0.993 | 146.91 |

A Bayes factor of greater than 50 at a given site was considered to be strong support for positive selection.

*Codons in the region interacting with Nef (SIV) is indicated in bold.

**Posterior probability for positive selection (*d*_N_>*d*_S_) at the site.
